# MicroRNA-10b promotes migration and invasion through Hoxd10 in human gastric cancer

**DOI:** 10.1186/s12957-015-0673-8

**Published:** 2015-08-28

**Authors:** Yuan-Yu Wang, Li Li, Zai-Yuan Ye, Zhong-Sheng Zhao, Zhi-Long Yan

**Affiliations:** Departments of Gastrointestinal Surgery and Pathology, Zhejiang Provincial People’s Hospital, Hangzhou, 310014 People’s Republic of China; Key Laboratory of Gastroenterology of Zhejiang Province, Hangzhou, 310014 Zhejiang People’s Republic of China; Departments of Pathology, Zhejiang Provincial People’s Hospital, Hangzhou, 310014 Zhejiang People’s Republic of China

**Keywords:** Gastric cancer, miR-10b, Hoxd10, Proliferation, Migration, Invasion

## Abstract

**Background:**

This study aims to investigate the effect of miR-10b overexpression on cancer cell proliferation, migration, invasion, and Hoxd10 expression.

**Methods:**

The effect of miR-10b on proliferation, migration, and invasion of MKN-28, BGC-823, and SGC-7901 cells and the expression of Hoxd10 protein in SGC-7901 and BGC-823 cells were detected following transfection of miR-10b inhibitor or Negative Control B. Expression of Hoxd10 protein in 436 paraffin-embedded cancer tissues was also investigated.

**Results:**

miR-10b was significantly upregulated in AGS, MKN-28, BGC-823, HCG-27, SGC-7901, and MKN-45 cell lines, miR-10b inhibitor significantly inhibited proliferation and migration of MKN-45, BGC-823 and SGC-7901 cells 48 h after transfection, while Hoxd10 protein in these cells lines had increased 72 h after transfection. Hoxd10 was highly expressed in gastric cancer and correlated with size of tumor, Lauren classification, depth of invasion, lymph node and distant metastasis, Tumor-Node-Metastasis (TNM) stage, and prognosis.

**Conclusions:**

miR-10b promotes migration and invasion through Hoxd10 in human gastric cancer cell lines and may play an important role in tumorigenesis, progression, and prognosis.

## Background

MicroRNAs (miRNAs) are a family of 21-to 25-nucleotide, noncoding small RNAs that primarily function as gene regulators by regulating mRNA translation and degradation [1]. miRNAs play a role in cancer or cancer prevention by adjusting the expression of downstream mRNA [2]. Previous studies show that miR-10b is upregulated in liver, pancreatic, and esophageal cancer, breast cancer with distant metastasis, and glioma tissues, and its expression is closely related with tumor progression [3–7]. miR-10b promotes migration and invasion through HOXD10, CADM1, MAPRE1, and KLF4 et al. [5–10], but the exact regulatory pathway is still poorly understood. However, nothing is known about the role of miR-10b gastric cancer metastasis. Therefore, in this study, we analyzed the level of miR-10b in human gastric cancer cell lines AGS, MKN-28, BGC-823, HCG-27, SGC-7901, 9811P, and MKN-45 and in the non-malignant gastric epithelial cell line GES-1, and the effect of miR-10b expression on gastric cancer cell proliferation, invasion, and migration. We aimed to elucidate the role of miR-10b in gastric cancer formation, development, invasion and metastasis, and its mechanism.

## Methods

### Cell culture

Human gastric cancer cell lines AGS, MKN-28, BGC-823, HCG-27, SGC-7901, 9811P, and MKN-45 and non-malignant gastric epithelial cell line GES-1 were obtained from Key Laboratory of Gastroenterology of Zhejiang Province (Hangzhou, China) and cultured in RPMI1640 containing 10 % fetal bovine serum (FBS), 50 U/ml penicillin, and 50 μg/ml streptomycin. All cells were maintained at 37 °C under an atmosphere of 5 % CO_2_.

### Archived gastric tissue samples and non-tumor mucosa

Gastric cancer tissues were collected from gastrectomy specimens from 436 patients (311 male, 125 female, median age 60.0 years, range 30–91) from the Department of Surgery, Zhejiang Provincial People’s Hospital, from January 1998 to January 2004. Tissues were formalin-fixed, paraffin-embedded, and diagnosed clinically and histopathologically at the Departments of Gastrointestinal Surgery and Pathology. The follow-up deadline was December 2008. The survival time was calculated from the date of surgery to the follow-up deadline or date of death, which was mainly due to carcinoma recurrence or metastasis. There were 55, 163, and 218 cases from the cardia, body, and antrum, respectively. According to the 2002 WHO histological classification of gastric carcinoma, there were 326 tubular adenocarcinomas, 16 papillary adenocarcinomas, 29 mucinous adenocarcinomas, and 65 signet-ring cell carcinomas. Thirteen were classified as highly differentiated adenocarcinomas, 128 as well or moderately differentiated adenocarcinomas, 293 as poorly differentiated and 2 as undifferentiated adenocarcinomas. There were 61 cases with distant metastasis. Ninety cases were categorized as stage I, 104 as stage II, 173 as stage III, and 69 as stage IV. Ninety-two noncancerous human gastric tissues were obtained from gastrectomies of adjacent gastric cancer margins greater than 5 cm. Routine chemotherapy was given to the patients with advanced stage disease after surgery, but no radiation treatment was administered to any patients included in our study.

### RT-PCR quantification of miR-10b

RT-PCR was performed to determine the expression of miR-10b. Briefly, total RNA was extracted from human gastric cancer cell lines AGS, MKN-28, BGC-823, HCG-27, SGC-7901, 9811P, MKN-45 and non-malignant gastric epithelial cell line GES-1 using Trizol (Invitrogen) according to the manufacturer’s instructions. cDNA synthesis was carried out with the miScript Reverse Transcription Kit (Qiagen). The specific reverse primer for miR-10b and U6 were provided by Qiagen. The resulting cDNA was amplified with the QuantiTect SYBR Green PCR Master Mix (Qiagen) using ABI 7500 FAST Real-time PCR (Applied Biosystems). PCR parameters were as follows: 95 °C for 15 min, followed by 40 cycles of 94 °C for 15 s, 55 °C for 30 s, 72 °C for 34 s. At the end of the PCR cycles, melting curve analysis was performed. The expression of miR-10b in cancer tissues was compared to matched normal samples using the 2^-△△CT^ method, and the expression of miR-10b in gastric cancer cells was compared to GES-1.

### miR-10b transfection

miR-10b inhibitor (miRCURY LNA™ microRNA Power inhibitor, 5 nmol, 5′-fluorescein labeled, Exiqon) and Negative Control B (miRCURY LNA™ microRNA Power Inhibitor Negative Control B, 5 nmol, 5′-fluorescein labeled, Exiqon) were transfected into MKN-28, BGC-823, and SGC-7901 cells grown in six-well dishes (plated at 5.0 × 10^5^ cells per well 24 h before transfection). Transfection was performed with Lipofectamine 2000 (Invitrogen). Twenty-four hours after, transfection cells were assayed for migration, invasion, and proliferation.

### Cell proliferation assay

The effect of miR-10b on proliferation of gastric cancer cells was evaluated by MTT assays. MKN-28, BGC-823, and SGC-7901 cells were seeded into 96-well plates at a density of 6.0 × 10^3^ cells/well in quintuplicate and allowed to adhere overnight. Ten microliters of MTT (5 mg/ml) (Sigma) was added to each well 48 or 72 h after infection, and cells were incubated for a further 4 h. Media was then removed, and 150 μl DMSO was added. Absorbance (A) at 570 and 630 nm were measured using a microplate reader. Relative cell proliferation (%) was shown by the following equation: Relative proliferation rate (%) = (A_570nm_–A_630nm_) of study group/(A_570nm_–A_630nm_) of control group × 100 %.

### In vitro cell migration and invasion assays

Twenty-four hours after infection, cells were harvested and subjected to the following assays. For migration assays, infected cells (6 × 104) were plated in triplicate in the top chamber of Transwells (Millicell Hanging Cell Culture Inserts, PIEP12R48, Millipore Corporation) with a membrane containing 8-μm diameter pores in 300 μl serum-free RPMI1640. The inserts were then placed into the bottom chamber wells of a 24-well plate containing RPMI1640 with 20 % FBS as a chemo-attractant. After 24 h of incubation, cells remaining on the insert top layer were removed by cotton swab scrubbing, and cells on the lower surface of the membrane were fixed in 100 % methanol for 15 min, followed by staining with Giemsa solution. Cell numbers in five random fields (400×) were counted for each chamber and the average values were calculated.

For invasion assays, infected cells (1.5 × 105) were plated in the top chamber with Matrigel-coated membrane (QCM ECMatrix Cell Invasion Assay, Millipore Corporation), whereas the bottom chambers were filled with conditioned medium. After 48 h incubation, migrated cells (lower side of the membrane) were counted as described above.

### Immunoblotting

Cells were lysed in RIPA buffer (150 mM NaCl, 10 mM Tris, pH 7.5, 1 % NP40, 1 % deoxycholate, 0.1 % SDS, protease inhibitor cocktail (Roche)). Total proteins were fractionated using the NuPAGE 4–12 % Bis-Tris gradient gel (Invitrogen) and transferred onto PVDF membrane. Membranes were blocked with 5 % non-fat milk in PBS/Tween-20 and incubated with antibodies against Hoxd10 (1:200, Santa Cruz) and β-actin (1:10,000, Abcam).

### Immunohistochemistry

Immunohistochemical analysis was performed as previously described [10]. Sections were incubated with mouse anti-Hoxd10 (1:50; Santa Cruz) overnight at 4 °C. The degree of immunostaining was reviewed and scored independently by two observers based on the proportion of positively stained tumor cells and intensity of staining. Tumor cell proportion was scored as follows: 0 (≤5 % positive tumor cells), 1 (6–25 % positive tumor cells), 2 (26–50 % positive tumor cells), and 3 (>51 % positive tumor cells). Staining intensity was graded according to the following criteria: 0 (no staining), 1 (weak staining = light yellow), 2 (moderate staining = yellow brown), and 3 (strong staining = brown). Staining index was calculated as the product of staining intensity score and the proportion of positive tumor cells. Using this method of assessment, we evaluated Hoxd10 expression in benign gastric epithelia and malignant lesions by determining the staining index with scores of 0, 1, 2, 3, 4, 6, or 9. The cutoff value for high and low expression level was chosen based on a measure of heterogeneity using the log-rank test with respect to overall survival. An optimal cutoff value was identified: a staining index score of ≥4 was used to define tumors with high Hoxd10 expression and a staining index score of ≤3 was used to indicate low Hoxd10 expression.

### Statistical analysis

All statistical analyses were performed using SPSS 16.0 software. Data were analyzed using Student’s *t* test, while categorical data were studied using the *χ*^2^ or Fisher exact test. Survival curves were estimated using the Kaplan-Meier method, and the log-rank test was used to calculate differences between the curves. Multivariate analysis using the Cox proportional hazards regression model was performed to assess the prognostic values of protein expression. Correlation coefficients between protein expression and clinicopathological findings were estimated using the Pearson correlation method. Statistical significance was set at *P* < 0.05.

## Results

### miR-10b is upregulated in gastric cancer cell lines

**Fig. 1:**
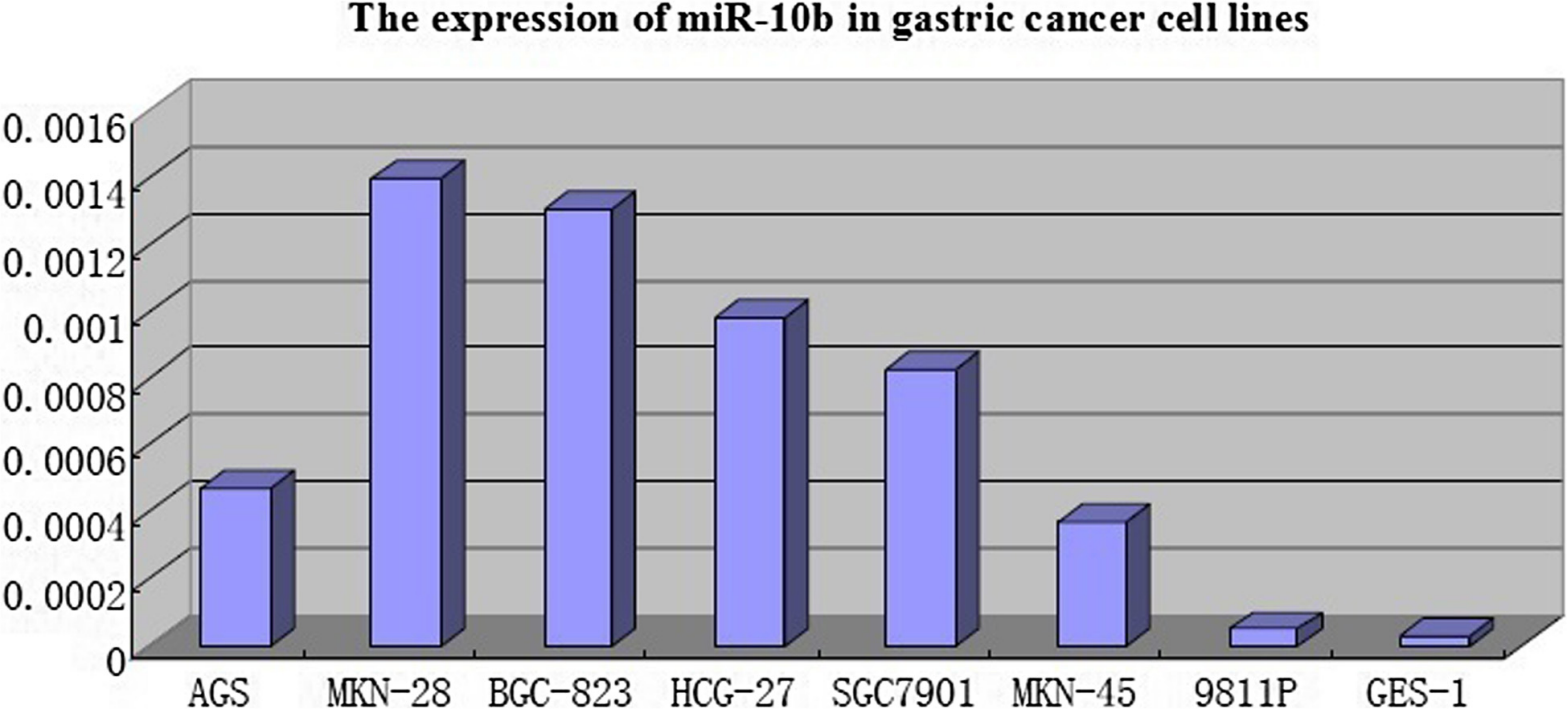
miR-10b is upregulated in gastric cancer cell lines

miR-10b was significantly upregulated in AGS, MKN-28, BGC-823, HCG-27, SGC-7901, and MKN-45 cell lines (4.7 × 10^−4^, 1.4 × 10^−3^, 1.3 × 10^−3^, 9.8 × 10^−4^, 8.3 × 10^−4^, and 3.7 × 10^−4^, respectively) compared with the non-malignant gastric epithelial cell line GES-1 (3.2 × 10^−5^) (*P* < 0.05). The expression of miR-10b did not significantly differ between 9811P cells (4.99 × 10^−5^) and non-malignant GES-1 cells (3.2 × 10^−5^) (*P* > 0.05) (Fig. 1).

### miR-10b inhibited cell proliferation, migration, and invasion in vitro

To study the biological role of miR-10b in gastric cancer, miR-10b inhibitor or Negative Control B was transfected into MKN-28, BGC-823, and SGC-7901 cells, and miR-10b levels were evaluated by RT-PCR. miR-10b levels in SGC-7901, BGC-823, and MKN-28 cells transfected with miR-10b inhibitor were much lower than in Negative Control B-infected cells (*P* < 0.05), 24 h after transfection.

Then, we evaluated the effect of miR-10b on the proliferation of gastric cancer cell lines using MTT assays. miR-10b inhibitor significantly inhibited proliferation of MKN-45, BGC-823, and SGC-7901 cells 48 h after transfection (*P* < 0.05). miR-10b inhibitor significantly inhibited proliferation of BGC-823 cells 72 h after transfection (*P* < 0.05) but only had a slight effect on the proliferation of MKN-45 and SGC-7901 cells (*P* > 0.05).

**Fig. 2:**
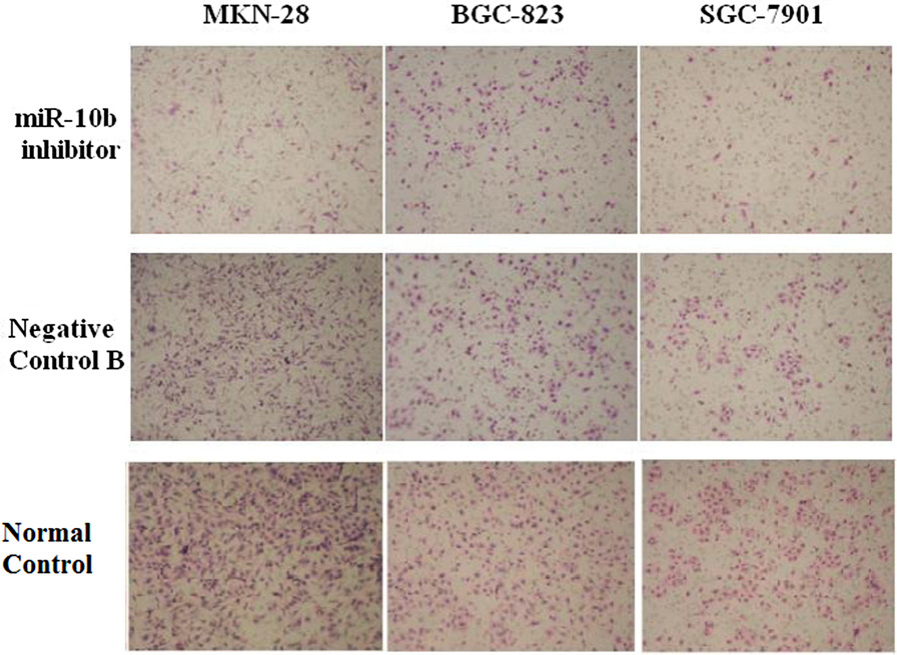
miR-10b inhibits cell migration. Representative fields of migrated cells on membrane

We further analyzed the effect of miR-10b on the migratory and invasive behavior of gastric cell lines (Table 1, Fig. 2). Gastric cancer cells infected with miR-10b inhibitor displayed significantly lower transmembrane migration capacity compared with those infected with Negative Control B cells and Normal Control cells 48 h after transfection (*P* < 0.05). No gastric cancer cell lines transfected with miR-10b inhibitor and Negative Control B invaded through the membrane of QCM ECMatrix Cell Invasion Assay 72 h after transfection.

**Table 1:** The number of migratory cells after infected with miR-10b inhibitor

	MKN-28	BGC-823	SGC-7901
miR-10b inhibitor	48.8 ± 3.96	54.2 ± 3.19	35.4 ± 2.07
Negative Control B	98.8 ± 2.59	77.4 ± 2.07	56.2 ± 2.77
Normal Control	107.4 ± 5.27	79.4 ± 3.65	60.2 ± 3.35
*t*值^a^	23.62	13.62	13.43
*t*值^b^	19.87	11.62	14.09
*P*值	0.0001	0.0001	0.0001

^a^miR-10b inhibitor vs Negative Control B

^b^miR-10b inhibitor vs Normal Control

### miR-10b directly regulated Hoxd10

**Fig. 3:**
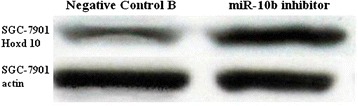
The level of Hoxd10 protein in SGC-7901 cells with miR-10b inhibitor-infected or Negative Control B-infected

**Fig. 4:**
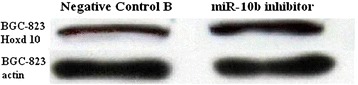
The level of Hoxd10 protein in BGC-823 cells with miR-10b inhibitor-infected or Negative Control B-infected

To understand the mechanisms by which miR-10b induces tumor invasion and metastasis, Western blotting for Hoxd10 in SGC-7901 and BGC-823 cells was performed at 72 h post-transfection. Hoxd10 expression was much higher in cells transfected with miR-10b inhibitor than in Negative Control B-infected cells (Figs. 3 and 4).

### Expression of Hoxd10 in gastric cancer and clinicopathological features and prognosis

**Fig. 5:**
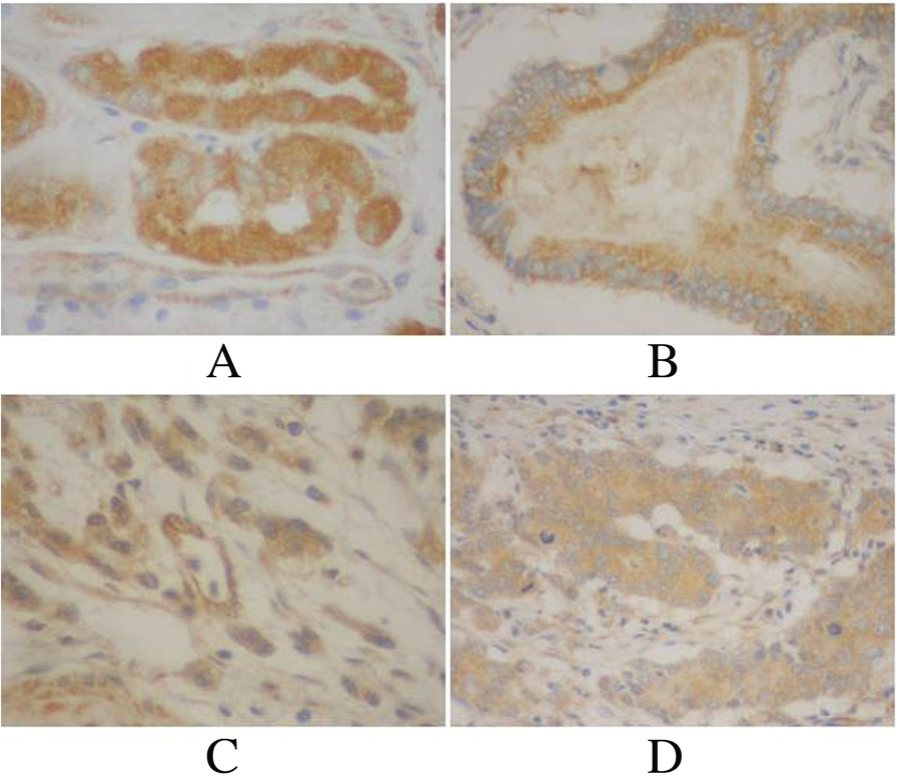
Immunohistochemical staining for Hoxd10 in gastric cancer lesions and noncancerous tissues, magnification 400×. **a** Hoxd10 was highly expressed in noncancerous tissues. **b** Hoxd10 was highly expressed in tubular adenocarcinoma. **c** Hoxd10 was highly expressed in poorly differentiated adenocarcinoma. **d** Hoxd10 was highly expressed in poorly differentiated adenocarcinoma.

**Fig. 6:**
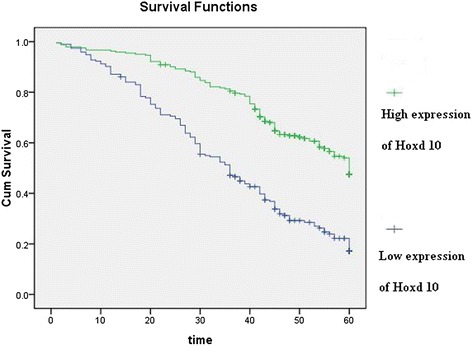
Kaplan-Meier curves with univariate analyses (log-rank) for patients with simultaneously low Hoxd10 expression versus high Hoxd10 expression tumors in all gastric cancer

**Fig. 7:**
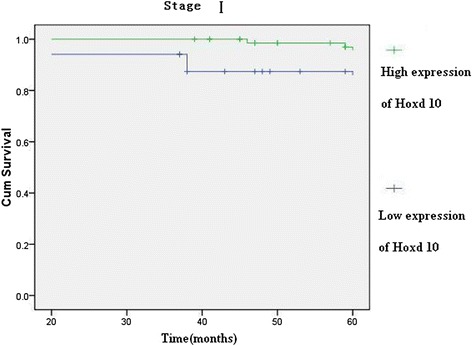
Kaplan-Meier curves with univariate analyses (log-rank) for patients with low Hoxd10 expression versus high Hoxd10 expression tumors in all gastric cancer in stage I

**Fig. 8:**
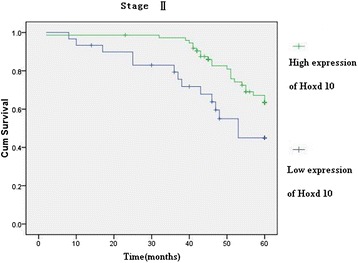
Kaplan-Meier curves with univariate analyses (log-rank) for patients with low Hoxd10 expression versus high Hoxd10 expression tumors in all gastric cancer in stage II

**Fig. 9:**
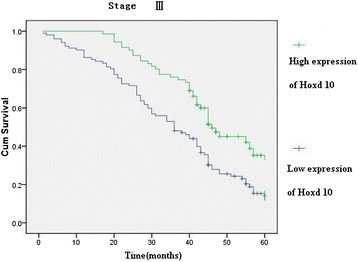
Kaplan-Meier curves with univariate analyses (log-rank) for patients with low Hoxd10 expression versus high Hoxd10 expression tumors in all gastric cancer in stage III

**Fig. 10:**
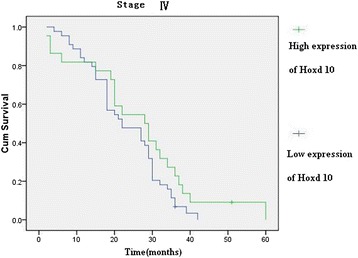
Kaplan-Meier curves with univariate analyses (log-rank) for patients with low Hoxd10 expression versus high Hoxd10 expression tumors in all gastric cancer in stage IV

Hoxd10 was upregulated in 82 (89.13 %) normal gastric mucosa samples (Fig. 5), and in 242 of 436 (55.5 %) cases of gastric cancer (Fig. 5). High expression of Hoxd10 correlated with size of tumor, Lauren classification, depth of invasion, lymph node and distant metastasis, and TNM stage (Table 2). We also analyzed the relationship between Hoxd10 expression and prognosis. The cumulative 5-year survival rate was 53.7 % in the high Hoxd10 protein expression group but was only 24.7 % in the low expression group (*χ*^2^ = 60.81, *P* = 0.001) (Fig. 6). In stages I, II, and III, the 5-year survival rate of patients with high expression of Hoxd10 was significantly higher than those in patients with low expression. In stage I, the cumulative 5-year survival rate was 91.8 % in the high Hoxd10 expression group but was only 82.4 % in low expression group (*χ*^2^ = 4.25, *P* = 0.039) (Fig. 7). In stage II, the cumulative 5-year survival rate was 68.9 % in high Hoxd10 expression group but was only 53.3 % in low expression group (*χ*^2^ = 5.35, *P* = 0.021) (Fig. 8). In stage III, the cumulative 5-year survival rate was 40.8 % in high Hoxd10 expression group but was only 15.7 % in low expression group (*χ*^2^ = 12.87, *P* = 0.0001) (Fig. 9). In stage IV, the expression of Hoxd10 did not correlate with the 5-year survival rate, the cumulative 5-year survival rate was 4.5 % in high Hoxd10 expression group but was only 2.3 % in low expression group in stage IV (*χ*^2^ = 2.19, *P* = 0.138) (Fig. 10). The factors with possible prognostic effects in gastric carcinoma were analyzed by Cox regression analysis. The study revealed that lymph node (*P* = 0.012) and distant metastases (*P* = 0.008), TNM stage (*P* = 0.001), and expression of Hoxd10 (*P* = 0.008) were independent prognostic factors in patients with gastric carcinoma. However, age, sex, tumor location and size, histological classification, tumor differentiation, Lauren’s classification, and regional lymph node stage had no prognostic value.

**Table 2:** Relationship of Hoxd10 expression with pathological parameters of tumor

Clinical parameters	HOXD10			
	Low	High	*t*/*χ* ^2^	*P*
Age (years)	59.77 ± 12.44	58.48 ± 11.85	1.107	0.269
Gender			0.519	0.4771
Male	135 (43.4 %)	176 (56.6 %)		
Female	59 (47.2 %)	66 (52.8 %)		
Location			4.379	0.112
Proximal	31 (56.4 %)	24 (43.6 %)		
Middle	74 (45.4 %)	89 (54.6 %)		
Distal	89 (40.8 %)	129 (59.2 %)		
Size			25.716	0.0001
<5 cm	88 (34.4 %)	168 (65.6 %)		
≥5 cm	106 (58.9 %)	74 (41.1 %)		
Lauren classification			63.165	0.0001
Intestinal	58 (26.0 %)	165 (74.0 %)		
Diffuse	136 (63.8 %)	77 (36.2 %)		
Histology classification			7.343	0.062
Papillary adenocarcinoma	3 (18.8 %)	13 (81.2 %)		
Tubular adenocarcinoma	142 (43.6 %)	184 (56.4 %)		
Mucinous adenocarcinoma	17 (58.6 %)	12 (41.4 %)		
Signet-ring cell carcinoma	32 (49.2 %)	33 (50.8 %)		
Histologic differentiation			5.25	0.154
Well	3 (23.1 %)	10 (76.9 %)		
Moderately	50 (39.1 %)	78 (60.9 %)		
Poorly	140 (47.8 %)	153 (52.2 %)		
Others	1 (50.0 %)	1 (50.0 %)		
Invasion depth			33.892	0.0001
T1	9 (15.8 %)	48 (84.2 %)		
T2	39 (35.8 %)	70 (64.2 %)		
T3	129 (52.9 %)	115 (47.1 %)		
T4	17 (65.4 %)	9 (34.6 %)		
TNM stages			60.859	0.0001
I	17 (18.9 %)	73 (81.1 %)		
II	30 (28.8 %)	74 (71.2 %)		
III	102 (59.0 %)	71 (41.0 %)		
IV	45 (65.2 %)	24 (34.8 %)		
Lymphatic metastasis			45.165	0.0001
No	40 (24.1 %)	126 (75.9 %)		
Yes	154 (57.0 %)	116 (43.0 %)		
Regional lymph nodes			51.95	0.0001
PN0	40 (24.1 %)	126 (75.9 %)		
PN1	67 (49.3 %)	69 (50.7 %)		
PN2	65 (65.7 %)	34 (34.3 %)		
PN3	22 (62.9 %)	13 (37.1 %)		
Distant metastasis			14.821	0.0001
No	153 (40.8 %)	222 (59.2 %)		
Yes	41 (67.2 %)	20 (32.8 %)

## Discussion

Recent evidence has shown that altered patterns of microRNA (miRNA) expression correlate with various human cancers. miR-10b was recently reported to be dysregulated in some types of cancer and to play a role in invasion and metastasis. Single microRNA (miRNA) can regulate expression of several or multiple principal targets in a specific microenvironment. In different cellular contexts, the same miRNA may exhibit diverse functions, depending on the repertoire and stoichiometry of its direct mRNA targets [11]. For instance, in breast cancer, microRNA-10b (miR-10b) promotes invasion and metastasis of tumor cells through regulation of HOXD10, E-cadherin, and syndecan-1 [12, 13]. miR-10b may positively regulate the invasion and metastasis of hepatocellular carcinoma through targeting CADM [8]. miR-10b induces glioma cell invasion by modulating MMP-14 and uPAR expression via HOXD10 [14].

The oncogenic or tumor suppressive effects of miR-10b might be tissue specific. Some debate exists on whether miR-10b is upregulated or downregulated in gastric cancer [9, 15]. Our previous study found miR-10b to be significantly upregulated in tissues, overexpression of miR-10b in gastric cancer tissues was associated with lymph node and distant metastases, and the results suggest that miR-10b may play an important role in gastric tumorigenesis, progression, and prognosis [16]. However, effects and potential mechanisms of action of miR-10b in the metastasis of gastric cancer have not been explored thoroughly. Here, we discuss a unique role of miR-10b in gastric cancer cell invasion and metastasis. In this study, we analyzed the level of miR-10b in human gastric cancer cell lines AGS, MKN-28, BGC-823, HCG-27, SGC-7901, 9811P, and MKN-45 and in the non-malignant gastric epithelial cell line GES-1, and the effect of miR-10b expression on gastric cancer cell proliferation, invasion, and migration. miR-10b was significantly upregulated in AGS, MKN-28, BGC-823, HCG-27, SGC-7901, and MKN-45 cell lines compared with the non-malignant gastric epithelial cell line GES-1. Liu et al. found miR-10b was highly expressed in poorly differentiated gastric cancer cell lines, such as BGC-823 and MKN45 [9]. We further analyzed the effect of miR-10b on the migratory and invasive behavior of gastric cell lines (BGC-823, SGC-7901, and MKN-45). miR-10b inhibitor significantly inhibited proliferation and migration and also significantly increased the level of Hoxd10 protein in SGC-7901 and BGC-823 cells. Some debate exists on whether miR-10b is inhibited or increased migration and invasion in gastric cell lines. miR-10b inhibitor increased both cell migration and invasion [17]. Inhibition of miR-10b activity by miR-10b inhibitor had no obvious effects on migration of MNK45 cells, while severely blocked cell invasion ability [9].

miR-10b can stimulate the upregulation of RhoC and AKT phosphorylation through targeting HOXD10, thus promoting cell invasion in gastric tumors [9]. There was no literature about the relationship between expression of HOXD10 and prognosis of patients. We further analyzed the association between Hoxd10 expression and clinicopathological features and prognosis. Hoxd10 was overexpressed in gastric cancer and correlated with size of tumor, Lauren classification, depth of invasion, lymph node and distant metastasis, TNM stage, and prognosis.

## Conclusions

In conclusion, we analyzed the expression of miR-10b in gastric cancer cell lines, and the effect of miR-10b on cell proliferation, migration, invasion assays, and Hoxd10 expression. Results reported here increase the understanding of the molecular basis of miR-10b in gastric cancer and suggest that miR-10b promotes migration and invasion through Hoxd10 in human gastric cancer cell lines. miR-10b may be a useful molecular biomarker for assessing the risk of gastric cancer development, and modulation of miR-10b may represent a therapeutic approach for treating gastric cancer.

## Abbreviations

AKT: V-akt murine thymoma viral oncogene homolog 1
CADM1: cell adhesion molecule 1
HOXD10: homeobox D10
KLF4: Kruppel-like factor 4
MAPRE1: microtubule-associated protein, RP/EB family, member 1
MMP-14: Matrix metallopeptidase 14
RhoC: Ras homolog family member C
TNM: Tumor-Node-Metastasis
uPAR: plasminogen activator, urokinase receptor
